# Dissemination of NDM-1-Producing Enterobacteriaceae Mediated by the IncX3-Type Plasmid

**DOI:** 10.1371/journal.pone.0129454

**Published:** 2015-06-05

**Authors:** Qing Yang, Lanfang Fang, Ying Fu, Xiaoxing Du, Yuqin Shen, Yunsong Yu

**Affiliations:** 1 State Key Laboratory for Diagnosis and Treatment of Infectious diseases, Collaborative Innovation Center for Diagnosis and Treatment of Infectious Diseases, The First Affiliated Hospital, College of Medicine, Zhejiang University, Hangzhou, Zhejiang, China; 2 Department of Infectious Diseases, First Affiliated Hospital, College of Medicine, Zhejiang University, Hangzhou, Zhejiang, China; 3 Department of Infectious Diseases, Sir Run Run Shaw Hospital, College of Medicine, Zhejiang University, Hangzhou, Zhejiang, China; Cornell University, UNITED STATES

## Abstract

The emergence and spread of NDM-1-producing Enterobacteriaceae have resulted in a worldwide public health risk that has affected some provinces of China. China is an exceptionally large country, and there is a crucial need to investigate the epidemic of *bla*_NDM-1_-positive Enterobacteriaceae in our province. A total of 186 carbapenem-resistant Enterobacteriaceae isolates (CRE) were collected in a grade-3 hospital in Zhejiang province. Carbapenem-resistant genes, including *bla*_KPC_, *bla*_IMP_, *bla*_VIM_, *bla*_OXA-48_ and *bla*_NDM-1_ were screened and sequenced. Ninety isolates were identified as harboring the *bla*_KPC-2_ genes, and five *bla*_NDM-1_-positive isolates were uncovered. XbaI-PFGE revealed that three *bla*_NDM-1_-positive *K*. *pneumoniae* isolates belonged to two different clones. S1-PFGE and southern blot suggested that the *bla*_NDM-1_ genes were located on IncX3-type plasmids with two different sizes ranging from 33.3 to 54.7 kb (n=4) and 104.5 to 138.9 kb (n=1), respectively, all of which could easily transfer to *Escherichia coli* by conjugation and electrotransformation. The high-throughput sequencing of two plasmids was performed leading to the identification of a smaller 54-kb plasmid, which had high sequence similarity with a previously reported pCFNDM-CN, and a larger plasmid in which only a 7.8-kb sequence of a common gene environment around *bla*_NDM-1_ (*bla*_NDM-1_-*trpF*- *dsbC*-*cutA1*-*groEL*-Δ*InsE*,) was detected. PCR mapping and sequencing demonstrated that four smaller *bla*_NDM-1_ plasmids contained a common gene environment around *bla*_NDM-1_ (IS*5*-*bla*_NDM-1_-*trpF*- *dsbC*-*cutA1*-*groEL*). We monitored the CRE epidemic in our hospital and determined that KPC-2 carbapenemase was a major risk to patient health and the IncX3-type plasmid played a vital role in the spread of the *bla*_NDM-1_ gene among the CRE.

## Introduction

Over the past ten years, carbapenemase-producing Enterobacteriaceae have disseminated rapidly throughout the world, posing an urgent threat to public health [1]. NDM-1 (New Delhi metallo-β-lactamase-1), one of the most serious carbapenemases, was first identified in *Klebsiella pneumoniae* and *Escherichia coli* in 2009, as NDM-1-producing Enterobacteriaceae rapidly disseminated, creating a global health crisis [2,3]. In China, Ho *et al*. first reported the emergence of *bla*_NDM-1_-positive *E*. *coli*, which was isolated from a one-year-old infant and his mother [4]. The epidemic dissemination of NDM-1-producing Enterobacteriaceae spp. has been confirmed in many cities in Hunan, Guangdong, and Zhejiang, as well as in areas of Hong Kong and Tai Wan [4–8]. At the beginning of the epidemic, most of the strains were isolated from stool samples, which suggested that intestinal colonization had occurred [5]. Increasingly, the epidemic has been reported to contribute to the active transmission of serious infectious diseases [8].

Reports of NDM-1-producing Enterobacteriaceae isolates in China have markedly increased since last year and have shown a typical geographical distribution. Our previous study confirmed that *bla*_NDM-1_-postive Enterobacteriaceae were not detected in a nationwide survey in 2009; however, several recent studies screened its epidemic dissemination in 2013 and reported that it is often associated with serious infectious diseases in some areas of China [5,7–9]. China is an exceptionally large country, and it is necessary to continuously monitor the distribution of *bla*_NDM-1_-positive Enterobacteriaceae in cities and provinces.

One of the major reasons for the rapid dissemination of the *bla*_NDM-1_ gene is its plasmid location. Plasmid incompatibility (Inc) groups, such as IncA/C, IncF, IncL/M and IncHI1, are the most prevalent plasmid types in the United Kingdom, Australia, Canada and the United States [10–13]. Our previous studies and the work of Ho *et al*. have demonstrated that the epidemic incompatibility type of the *bla*_NDM-1_ plasmid in China is different from that in other countries: the IncX3-type was the most prevalent type harboring the *bla*_NDM-1_ gene [5,7]. In the present study, we explored the carbapenem-resistant genotypes among carbapenem-resistant Enterobacteriaceae isolates (CRE) isolates in a grade 3 hospital in Zhejiang province and focused on the epidemic factors and the dispersal mechanism of *bla*_NDM-1_-positive Enterobacteriaceae isolates.

## Materials and Methods

### Species identification, antimicrobial susceptibility and carbapenemase gene determination

Enterobacteriaceae isolates were collected in China in a grade-3 hospital in Hangzhou, Zhejiang province from January to September 2013. Species were identified using the VITEK 2 system (bioMérieux, Marcy l’ Etoile, France). The broth microdilution method was applied to test the minimal inhibitor concentrations (MICs) of the following eight antibiotics: ertapenem (ETP), imipenem (IMP), meropenem (MEM), cefepime (FEP), ceftazidime (CAZ), levofloxacin (LEV), amikacin (AMK) and tigecycline (TGC). The isolates that were non-susceptible to ertapenem (MIC≥ 0.5 mg/L) were selected and further screened for carbapenem-resistant genes, including *bla*_KPC_, *bla*_IMP_, *bla*_VIM,_ *bla*_OXA-48_ and *bla*_NDM-1_, by PCR followed by sequencing, as previously reported [14].

### Susceptibility testing of *bla*_NDM-1_-positive Enterobacteriaceae

The E-test method (AB bioMérieux, Sweden) was used to investigate the antibiotic profile of the *bla*_NDM-1_-positive Enterobacteriaceae isolates. The susceptibility breakpoints were interpreted according to the CLSI guideline. The U.S. Food and Drug Administration (FDA) breakpoint tigecycline criterion for Enterobacteriaceae was applied in our study (resistant breakpoint ≥2 mg/L) [15,16]. Standard strains (*E*. *coli* ATCC 25922 and *P*. *aeruginosa* ATCC27853) were used as susceptibility controls.

### Bacterial genotyping

To analyze the homology of the *bla*_NDM-1_-positive *K*. *pneumoniae* isolates, pulsed-field gel electrophoresis (PFGE) was used in our study. The genomic DNA of the *bla*_NDM-1_-positive *K*. *pneumoniae* isolates and reference marker *Salmonella* H9812 were digested by XbaI endonuclease, which was performed with a CHEF-Mapper XA PFGE system (Bio-Rad, USA) with a 5–35 s linear ramp for 22 h at 6 V/cm and 14°C [17]. The gel was stained with GelRed (Biotium, USA), according to the manufacturer’s instructions, and visualized using a 254 nm UV light. The PFGE profiles were analyzed with BioNumerics software (Applied Maths, Sint-Martens-Latern, Belgium).

### Determination of the *bla*_NDM-1_ gene location

The total bacterial DNA was first prepared in agarose plugs, digested with S1 nuclease and further separated by PFGE, as reported previously [9]. The DNA fragments were transferred horizontally to a nylon membrane (Millipore), hybridized with a digoxigenin-labeled *bla*_NDM-1_ probe and detected using a nitro-blue tetrazolium/5-bromo-4-chloro-3’-indolylphosphate color detection kit (Roche Applied Sciences).

A filter-mating experiment was performed between the *bla*_NDM-1_-positive isolates and *E*. *coli* J53 (an azide-resistant recipient). The successful transconjugants were selected on Mueller-Hinton agar incorporating ampicillin (50 mg/L) and azide (200 mg/L) and confirmed by PCR and sequencing to have the *bla*_NDM-1_ gene. The plasmid DNA of the *bla*_NDM-1_-positive Enterobacteriaceae isolates was extracted using a Plasmid DNA Midi kit (Qiagen, Germany), electrotransformed to *E*. *coli* DH5α competent cells and selected on Mueller-Hinton agar plates containing ampicillin (50 mg/L). The transconjugants and the transformants identified the species by the VITEK 2 system, tested the MICs using E-test strips and confirmed the resistant genes by PCR.

### Incompatibility typing of the *bla*_NDM-1_ plasmid

The plasmid classification methods developed by Carattoli *et al*. and Johnson *et al*. were applied in our study to determine the Inc type of the NDM-1-producing isolates [18,19]. S1-PFGE and Southern blot with the IncX3 probe were utilized to verify the Inc type of the *bla*_NDM-1_ plasmid.

### High-throughput sequencing of the *bla*_NDM-1_ plasmids

Plasmids DNA with different sizes were extracted using a Qiagen Plasmid DNA Midi Kit (Qiagen, Gemany) and were then sequenced using HiSeq 2000 (Illumina) technology following the 2×100bp paired-end protocol. The derived reads were trimmed and assembled using the CLC genomic workbench version 7.5 (CLC Bio, Aarhus, Denmark). Gaps between contigs were closed by PCR. Genome annotation was performed using the RAST (Rapid Annotation using Subsystems Technology) annotation website server (http://rast.nmpdr.org/rast/cgi) and the BLASTX algorithm (http://blast.ncbi.nlm.nih.gov).

### Genetic environment analysis of the *bla*_NDM-1_ gene

PCR mapping was applied to analyze the *bla*_NDM-1_ genetic structure. The PCR primers were designed according to the plasmid sequence of the 13500 strain and all PCR amplicons were sequenced (S1 Table).

### Nucleotide sequence accession numbers

The nucleotide sequences have been submitted to the GenBank database with the assigned accession numbers KR059865 (strain 13500) and KR059864 (strain 13450).

## Results

### Species identification, susceptibility results, clinical feature and carbapenem-resistant genotypes of the CRE

In total, 186 ETP non-susceptible Enterobacteriaceae isolates were studied (S2 Table), most of which were resistant to cephalosporin and levofloxacin as well. IMP and MEM showed relatively higher susceptibility than ETP. Tigecycline and amikacin were the two most effective agents against all the species.

Among the 186 CRE isolates, 90 isolates were KPC-2 carbapenemase producers, four isolates carried the *bla*_IMP-4_ gene, one *C*. *freundii* carried *bla*_IMP-1_, and 2 *K*. *pneumoniae* carried *bla*_KPC-2_ combined with *bla*_VIM-1_. Five NDM-1 carbapenemase-producing isolates (3 *K*. *pneumoniae*, 1 *E*. *coli* and 1 *E*. *cloacae*) were identified (S2 Table).

### Clinical features, susceptibility results and the PFGE profile of the *bla*_NDM-1_-positive Enterobacteriaceae isolates

**Fig 1:**
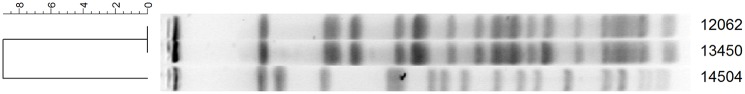
This is the Fig 1 PFGE analysis of NDM-1-producing *K*. *pneumoniae*.

Table 1 shows that five NDM-1 carbapenemase-producing Enterobacteriaceae were isolated from four patients, and two (13500 and 13450) were separated from the same person. The patients were elderly, with an average age of 75 years. Two patients were males with bloodstream infections. Three of the four patients were diagnosed as having a urinary tract infection. Five *bla*_NDM-1_-positive isolates that were susceptible to tigecycline and colistin were highly resistant to cephalosporins, carbapenems and aztreonam and were variably resistant to aminoglycosides, ciprofloxacin and minocycline (Table 2). Two PFGE profiles were identified among the *K*. *pneumoniae* isolates (clone A and clone B) (Table 1, Fig 1).

**Table 1:** Clinical features of the five *bla*_NDM-1_-positive isolates.

isolates	Species	Clinical feature						PFGE profile	*bla*_NDM-1_ plasmid size(kb)
		Age, years	Gender	Samplesource	Diagnosis	Ward	Outcome		
13500	*E*. *cloacae*	75	Male	Blood	Septicemia	Hepatobiliary and pancreatic surgery	Death	ND	33.3~54.7
13450	*K*. *pneumoniae*	75	Male	Blood	Septicemia	Hepatobiliary and pancreatic surgery	Death	Clone A	104.5~138.9
14504	*K*. *pneumoniae*	64	Female	Urine	Urinary tract infection	Hepatitis	Discharge	Clone B	33.3~54.7
12062	*K*. *pneumoniae*	93	Female	Urine	Urinary tract infection	VIP	Death	Clone A	33.3~54.7
11907	*E*. *coli*	67	Male	Blood	Urinary tract infection	Infectious diseases	Discharge	ND	33.3~54.7

ND: Not Detected.

**Table 2:** Antibiotic susceptibilities and β-lactamase detection of *bla*_NDM-1_-positive Enterobacteriaceae isolates, transformants and transconjugants.

Isolates	Antibiotics (ug/mL)														
	CTX	CAZ	SAM	TZP	CPS2/1	ATM	IPM	MEM	IPM/IPM+EDTA	CIP	GEN	AMK	MIN	CST	TGC
13500	>32	>256	>256	>256	>256	>256	>32	>32	256/<1	4	32	2	6	1	1
14504	>32	>256	>256	>256	>256	>256	>32	>32	256/<1	>32	2	1.5	24	4	2
13450	>32	>256	>256	>256	>256	>256	>32	>32	192/<1	0.38	64	24	4	1.5	1
12062	>32	>256	>256	>256	>256	>256	>32	>32	256/<1	0.5	64	12	3	4	1
11907	>32	>256	>256	>256	>256	>256	>32	>32	256/<1	>32	>256	>256	3	4	1
DH5a	0.016	0.064	6	0.75	0.032	0.032	0.125	0.023	<4/<1	0.012	0.19	0.5	0.5	0.125	0.19
13500-DH5a	>32	>256	>256	256	96	12	3	1.5	<4/<1	0.008	0.19	0.38	0.5	0.094	0.125
14504-DH5a	>32	≥256	>256	>256	>256	24	6	8	8/<1	0.023	0.25	0.38	1	0.125	0.19
13450-DH5a	>32	≥256	>256	>256	>256	24	12	4	6/<1	0.032	0.19	0.5	0.75	0.125	0.19
12062-DH5a	>32	≥256	>256	>256	>256	24	12	1.5	16/<1	0.032	0.38	0.38	1	1	0.38
11907-DH5a	>32	≥256	>256	>256	192	48	6	6	6/<1	0.032	0.25	0.38	1	0.5	0.125
J53	0.047	0.19	16	1.5	0.094	0.047	0.25	0.047	<4/<1	0.008	0.25	1	1	0.125	0.25
13500-J53	>32	>256	>256	>256	>256	>256	32	>32	<4/<1	4	32	2	3	0.19	0.25
14504-J53	>32	>256	>256	>256	128	12	4	6	<4/<1	0.008	0.19	0.75	1	0.25	0.25
13450-J53	>32	>256	>256	>256	>256	48	12	>32	<4/<1	0.008	1.5	2	1.5	0.125	0.25
12062-J53	>32	≥256	>256	>256	>256	32	≥32	32	48/<1	0.012	0.75	1.5	2	1	0.25
11907-J53	>32	>256	>256	>256	>256	>256	4	8	<4/<1	0.47	0.75	>2	1.5	0.5	0.38

CTX, cefotaxime; CAZ, ceftazidime; SAM, ampilillin/sulbactam; TZP, piperacillin/tazobactam; CPS2/1, cefoperazone/sulbactam 2:1; ATM, aztreonam; IPM, imipenem; MEM, meropenem; IPM/IPM+EDTA, imipenem/ imipenem+EDTA; CIP, ciprofloxacin; GEN, gentamicin; AMK, amikacin; MIN, minocycline; CST, colistin; TGC, tigecycline.

### Gene location of the *bla*_NDM-1_ and Inc-type of the *bla*_NDM-1_ plasmid

S1-PFGE followed by Southern blot confirmed that the *bla*_NDM-1_ genes among the five isolates were located on plasmids, and four of them were on the same size plasmid, which ranged from 33.3 to 54.7 kb, whereas one isolate was on a 104.5~138.9 kb plasmid. The filter mating experiments as well as the electrotransformation experiments further verified the transferability of these *bla*_NDM-1_ plasmids. Table 2 shows the susceptibility results of the recipient isolates, transconjugants and transformants.

**Fig 2:**
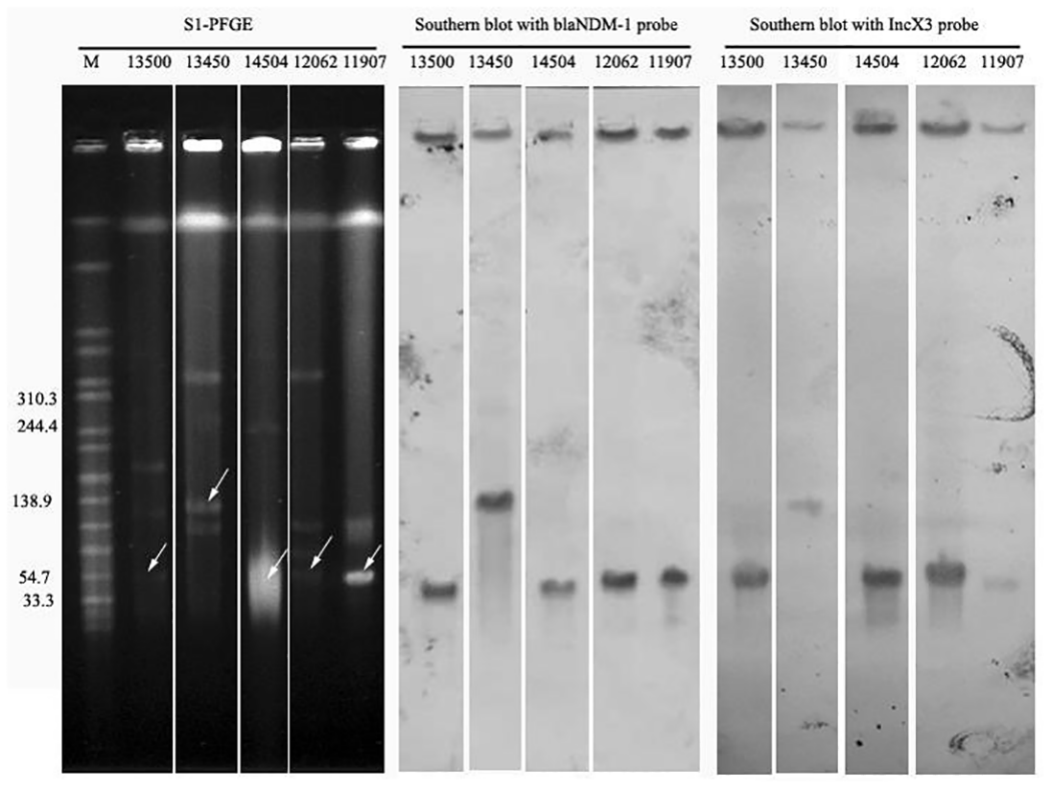
This is the Fig 2 Location of the *bla*_NDM-1_ gene and the IncX3 type of *bla*_NDM-1_ plasmid.

Multiple plasmids were identified with two incompatibility plasmid classification methods (data not shown), whereas the Southern blot result confirmed that all the *bla*_NDM-1_ plasmids belonged to the IncX3-type plasmid (Fig 2).

### Plasmid feature and genetic structure of the *bla*_NDM-1_ gene

According to the size difference, the *bla*_NDM-1_ plasmids in strain 13500 and strain 13450 were selected and fully sequenced. Sequence of the *bla*_NDM-1_ plasmids in strain 13500 was completed, which was 54,035bp in length and contained 53 open reading frames (ORFs), with an average GC content of 0.49%. A 7.8 kb plasmid fragment in strain 13450 was obtained by sequencing. Four of the isolates with similar plasmids contained a common genomic structure around the *bla*_NDM-1_ gene, which was composed of a cluster of genes, IS*5*-*bla*_NDM-1_-*trpF*-*dsbC*-*cutA1*-*groEL*, whereas the larger one (13450 strain) was missing the IS*5* upstream of the *bla*_NDM-1_.

## Discussion

With its global dissemination, NDM-1 carbapenemase-producing Enterobacteriaceae spp. has emerged and disseminated dramatically throughout China, causing serious infectious diseases and gaining wide governmental attention. In the present study, we undertook a systematic study of the epidemic status of CRE in our hospital and revealed that the KPC-producers were among the most serious problems in CRE isolates. We further investigated the genomic characteristics of *bla*_NDM-1_-positive Enterobacteriaceae. Our results suggested that the *bla*_NDM-1_ genes were located on an IncX3-type plasmid and presented a similar genetic sequence around the *bla*_NDM-1_, which indicated the potential threat in China of CRE induced by the IncX3 plasmid encoding NDM-1 carbapenemase in Zhejiang.

Resistance to carbapenems, which are known as the 'last resort' drugs to serious infections caused by Gram-negative bacteria, is becoming a critical problem. Although most clinical Enterobacteriaceae pathogens continue to show relatively high susceptibility to carbapenems, an increasing number of studies have reported the emergence of Enterobacteriaceae that are not susceptible to carbapenem [20,21], which may present a serious problem because tigecycline and colistin would be the only useful therapies against Enterobacteriaceae. Our previous study reported that Zhejiang was one of the provinces most affected by CRE pathogens in China, and KPC carbapenemase was directly attributable to the mediation of carbapenem resistance in *K*. *pneumoniae* [22,23]. Metallo-β-lactams, including IMP, VIM and NDM-1, contributed to carbapenem resistance in Enterobacteriaceae, which has caused global concern, particularly in recent years [24]. Enterobacteriaceae isolates producing OXA-48 carbapenemase, found in Europe, in southern and eastern areas of the Mediterranean region, and in Africa, have not been identified in China [25]. In our study, we investigated *bla*_KPC_, *bla*_VIM_, *bla*_OXA-48_, *bla*_IMP_ and *bla*_NDM-1_ and detected these resistant genes by PCR followed by sequencing technology.

NDM-1-producing Enterobacteriaceae isolates have been reported in many countries since being reported in China in 2011 [4]. A number of studies have reported the emergence and dissemination of NDM-1-producing Enterobacteriaceae isolates, one of which warned of the high incidence and endemic spread of NDM-1-producing Enterobacteriaceae in Henan, China [8]. This study reported that five Enterobacteriaceae isolates positive for the *bla*_NDM-1_ gene were endemic in our hospital last year and confirmed that all the *bla*_NDM-1_ genes were located on an IncX3-type plasmid. The emergence of the IncX3-type plasmid carrying the *bla*_NDM-1_ gene was first reported in 2012 in multiple cities in China by Ho *et al* [5]. We then identified a *C*. *freudii* isolate positive for the *bla*_NDM-1_ gene and further confirmed, by high-throughput sequencing technique, that it was another example of the IncX3-type plasmid carrying the *bla*_NDM-1_ gene (pCFNDM-CN, GenBank accession No. JX254913) [7]. We validated our concern that five Enterobacteriaceae isolates, including *E*. *cloacae*, *K*. *pneumoniae* and *E*. *col*, all of which belonged to the IncX3-type plasmid, carried the *bla*_NDM-1_ plasmid. Notably, by high-throughput plasmid sequencing technology, we confirmed that the plasmid sequence in the 13500 strain showed a high similarity to that of the pCFNDM-CN (99% similarity), which warned us that this type of IncX3-type plasmid has spread around our province, and it has been a threat in the dissemination of the *bla*_NDM-1_ gene among Enterobacteriaceae strains in China.

Clinical data confirmed that four patients from four wards in our hospital were associated with severe infectious diseases such as bloodstream infections and urinary tract infections. Additionally, we showed that four plasmids were of identical size, ranging from 33.3 to 54.7 kb, and their genetic structure was similar to that surrounding the *bla*_NDM-1_ gene: IS*5*-*bla*_NDM-1_-*trpF*-*dsbC*-*cutA1*-*groEL*. In addition, a larger IncX3 plasmid encoding the *bla*_NDM-1_ gene and ranging from 104.5~138.9 kb in size was found in a *K*. *pneumoniae* isolate (13450), which lacked the IS*5* upstream of *bla*_NDM-1_, suggesting that the larger plasmid may be a novel type meriting comprehensive attention.

We report, for the first time, the endemic spread of Enterobacteriaceae spp. with the *bla*_NDM-1_ gene in our hospital in Zhejiang. Our work adds to the hypothesis that the IncX3-type plasmid is a serious threat, particularly because of the global dissemination of NDM-1-producing Enterobacteriaceae spp. We strongly suggest that the issue of Enterobacteriaceae spp. harboring he IncX3- type plasmid containing the *bla*_NDM-1_ gene should receive national attention.

## Supporting Information

S1 Table This is the S1 Table Primers for PCR mapping. (DOCX)

S2 Table This is the S2 Table Susceptibility results and carbapenem-resistant genotype of the 186 Enterobacteriaceae isolates. (DOCX)
