# Supplementary material for: Dissemination of NDM-1-Producing Enterobacteriaceae Mediated by the IncX3-Type Plasmid
Source: PLoS One. 2015 Jun 5;10(6):e0129454. doi: 10.1371/journal.pone.0129454 (PMC4457825; doi:10.1371/journal.pone.0129454)
Supplement: S1 Table — (DOCX) [file pone.0129454.s001.docx]

**Table S1**. Susceptibility results and carbapenem-resistant genotype of the 186 Enterobacteriaceae isolates

| Test items | | *K. pneumoniae*  (118 isolates) | *E. coli*  (21 isolates) | *E. cloacae*  (16 isolates) | S. *marcescens*  (10 isolates) | *E. aerogenes*  (8 isolates) | *C. freundii*  (5 isolates) | *R. planticola*  (2 isolates) | Others  (6 isolates) |
| --- | --- | --- | --- | --- | --- | --- | --- | --- | --- |
| Susceptibility rate (%) | ETP | 0 | 0 | 0 | 0 | 0 | 0 | 0 | 0 |
|  | IPM | 11.9 | 57.1 | 68.8 | 20 | 25 | 80 | 0 | 33.3 |
|  | MEM | 9.3 | 52.4 | 68.8 | 30 | 12.5 | 80 | 0 | 16.7 |
|  | FEP | 5.9 | 9.5 | 37.5 | 10 | 25 | 0 | 0 | 50 |
|  | CAZ | 1.7 | 4.8 | 0 | 30 | 0 | 0 | 0 | 0 |
|  | LEV | 14.4 | 4.8 | 31.2 | 10 | 50 | 0 | 100 | 50 |
|  | AMK | 35.6 | 52.4 | 68.8 | 80 | 75 | 80 | 100 | 33.3 |
|  | TGC | 94.1 | 95.2 | 81.2 | 70 | 75 | 100 | 100 | 66.7 |
| Carbapenem resistant genotypes (%) | *bla*_KPC-2_ | 79 (66.9%) | 2 (9.5%) | 1 (6.3%) | 2 (20.0%) | 3 (37.5%) | 1 (20.0%) | 2 (100.0%) | ND |
|  | *bla*_IMP-1_ | ND | ND | ND | ND | ND | 1 (20.0%) | ND | ND |
|  | *bla*_IMP-4_ | 2 (1.7%) | 1 (4.8%) | 1 (6.3%) | ND | ND | ND | ND | ND |
|  | *bla*_NDM-1_ | 1 (0.8%) | 1 (4.8%) | 1 (6.3%) | ND | ND | ND | ND | ND |
|  | *bla*_KPC-2_+ *bla*_VIM-1_ | 2 (1.7%) | ND | ND | ND | ND | ND | ND | ND |
|  | *bla*_IMP-4_+*bla*_NDM-1_ | 2 (1.7%) | ND | ND | ND | ND | ND | ND | ND |
|  | Other unknowm mechanism | 32 (27.1%) | 17 (81%) | 13 (81.3%) | 8 (80%) | 5 (62.5%) | 3 (80%) | 0 (0%) | 6 (100%) |

ETP, Ertapenem ; IMP, Imipenem; MEM, Meropenem; FEP, Cefepime; CAZ, Ceftazidime; LEV, Levofloxacin; AMK, Amikacin; TGC, Tigecycline.
