# Supplementary material for: Dissemination of NDM-1-Producing Enterobacteriaceae Mediated by the IncX3-Type Plasmid
Source: PLoS One. 2015 Jun 5;10(6):e0129454. doi: 10.1371/journal.pone.0129454 (PMC4457825; doi:10.1371/journal.pone.0129454)
Supplement: S2 Table — (DOCX) [file pone.0129454.s002.docx]

**Table S2** Primers for PCR mapping

| Name | Seq (5’→3’) |
| --- | --- |
| A207-W3F | GTGTTAGTGGAGCCACTGAT |
| 17L-465 | CCGCAACCATCCCCTCTT |
| A207-W5F | CCAGCTCGCACCGAATGTCT |
| A207-W6F | GACAAGCTGCGCTGAGCCAT |
| A207-W4R | ATCGCTTCGGTGGTGATCAT |
